# Points for Members of Parliament

**Published:** 1919-02-22

**Authors:** 


### THE ETHICS OF CONTROVERSY.

### Points for Members of Parliament.

It will be remembered that a few weeks ago Sir Cooper Perry replied in the columns of the *Daily Telegraph* to the Secretary of the Royal British Nurses' Association, who alleged that he had informed a representative of that journal that the College Bill for the Registration of Nurses was "not contentious," and further that "but for his opposition and the opposition of those sitting on the boards of hospitals," a Registration Bill would have been passed many years ago.

Sir Cooper Perry dealt with these charges by stating that he had no communication whatever with any representative of the *Daily Telegraph*, had never made so foolish a statement as that the Bill was not contentious, and had himself never been opposed to the principle of State Registration. We now learn that the "tone" of this letter was objected to, and that the Secretary of the R.B.N.A. wrote a rejoinder to the Editor of the *Daily Telegraph*, which he "could not find space to insert," thus leaving the public under the painful impression that the lady in question had made statements she was unable to prove, a position obviously most unfair when he had been "supplied with irrefutable evidence to the contrary."

"We are now permitted to know what was the "irrefutable evidence" that the suppressed letter would have afforded, for in spite of an unsympathetic Editor, the Secretary of the R.B.N.A. has been able to get her letter printed elsewhere. She writes that she was led to her conclusions from the report of Sir Cooper Perry's statements and from his remarks at the Meeting in Wimpole Street, to believe that he regarded the College Bill as "not contentious." Thus the original allegation that he communicated with the *Daily Telegraph* correspondent is quietly dropped without any attempt at substantiation. Then comes the reply to his statement that he had never been opposed to the principle of State Registration. This is met by the suggestion, impertinent in every sense, that "the discovery of this fact a short time since is as new to himself as to us," and as a proof that Sir Cooper Perry is opposed to the principle of State Registration it is added that years ago he, with many others objected to the attempt of the British Nurses' Association to form, not a State Register at all, but a Register of its own, to be compiled at a time when those who had most to do with the training and examination of nurses had declined to take any part in it. No wonder that under such circumstances the Editor of the *Daily Telegraph* politely regretted that he could not find room for such "irrefutable evidence."

